# Towards kilohertz synchrotron coherent diffractive imaging

**DOI:** 10.1107/S1600576722003466

**Published:** 2022-05-08

**Authors:** Gerard N. Hinsley, Cameron M. Kewish, Grant A. van Riessen

**Affiliations:** a Department of Mathematical and Physical Sciences, School of Computing, Engineering and Mathematical Sciences, La Trobe University, Bundoora, Victoria 3086, Australia; b Australian Nuclear Science and Technology Organisation, Australian Synchrotron, Victoria 3168, Australia; c Melbourne Centre for Nanofabrication, Clayton, Victoria 3168, Australia

**Keywords:** coherent diffractive imaging, coherent X-ray imaging, kHz X-ray imaging, nanoscale dynamics, phase retrieval

## Abstract

X-ray coherent diffractive imaging (CDI) techniques have been applied with widespread impact to study nanoscale material properties. New fast framing detectors may reveal dynamics that occur at millisecond timescales. This work demonstrates by simulation that kilohertz synchrotron CDI is possible, by making use of redundant information from static parts of the image field. Reconstruction ambiguities are strongly suppressed by applying a spatio­temporal constraint, obviating the need for slower methods of introducing diversity such as ptychography. The relationship between image fidelity and time resolution is investigated and shows that dynamics an order of magnitude faster can be reconstructed, compared with conventional CDI.

## Abstract 2

This work shows how spatiotemporal redundancy can overcome the twin-image stagnation mode in coherent diffractive imaging, and explores the relationship between detector frame rate and signal-to-noise ratio in the application of imaging nanoscale dynamic behaviour at kHz frame rates.

## 1. Introduction

Coherent diffractive imaging (CDI) with X-rays [see *e.g.* Miao *et al.* (2015 ▸), and references therein] has attracted recent interest for its potential to achieve high-resolution imaging of nanoscale dynamic behaviour (Lo *et al.*, 2018 ▸; Takayama *et al.*, 2021 ▸), by taking advantage of new developments in fast hybrid pixel photon-counting detectors (Brönnimann & Trüb, 2020 ▸) such as the currently available EIGER (Dinapoli *et al.*, 2011 ▸) and diffraction-limited sources (Eriksson *et al.*, 2014 ▸). However, the robustness of CDI is limited by inherent ambiguities such as twin images in the image reconstruction (Fienup & Wackerman, 1986 ▸; Guizar-Sicairos & Fienup, 2012 ▸) and dependence on prior knowledge of the object or illumination. Ptychography overcomes these issues by introducing a high degree of information redundancy in the spatial domain from transverse translations of the object (Rodenburg & Faulkner, 2004 ▸) through a spatially limited illuminating probe with a high degree (typically >60%; Bunk *et al.*, 2008 ▸) of areal overlap between adjacent positions. This has been shown to allow constraints on the illumination and other experimental factors to be relaxed (Thibault *et al.*, 2008 ▸; Maiden & Rodenburg, 2009 ▸; Maiden *et al.*, 2012 ▸; Thibault & Menzel, 2013 ▸) and underpins developments such as fly scanning (Pelz *et al.*, 2014 ▸; Clark *et al.*, 2014 ▸; Jones *et al.*, 2021 ▸). Despite impressive advances in robustness and imaging rates, the achievable temporal resolution of dynamic ptychography is limited by interrelated factors of detector performance, the mechanical limitations to object scanning and the degree of redundancy in the diffraction data that is needed to reliably reconstruct real-space images. Objects which are unstable or which exhibit a high degree of dynamic behaviour introduce artefacts into the reconstruction. This forces *in situ* ptychography to be used in applications where objects undergo step-wise modification but can remain stable during relatively slow measurement (Kourousias *et al.*, 2016 ▸; Baier *et al.*, 2016 ▸).

Using redundancy in the time domain for the introduction of a constraint has recently been proposed in CDI (Lo *et al.*, 2018 ▸; Tao *et al.*, 2018 ▸), exploiting ‘overlap’ between successive images to achieve similar advantages to ptychography. An algorithm that exploits redundancy in time-series CDI by exploiting intentional or incidental spatiotemporal diversity in the diffraction data was recently reported (Hinsley *et al.*, 2020 ▸). This approach generates a spatiotemporal real-space constraint by segmenting time-dependent and time-independent regions of an object by iteratively refining an estimate of the dynamic behaviour in the object over a time series. It does not rely on the inclusion of static reference structures in either the object or illumination. This algorithm uses all of the diffraction information obtained from a dynamic object undergoing a transformation to suppress artefacts in the reconstructions (Hinsley *et al.*, 2020 ▸). This has important implications for studying systems that exhibit morphological changes in response to external stimuli (Kourousias *et al.*, 2016 ▸; Baier *et al.*, 2016 ▸; Auernhammer *et al.*, 2009 ▸; Kuo *et al.*, 2011 ▸; Sun & Wang, 2011 ▸; Zhao *et al.*, 2017 ▸) which are intrinsically unstable, or which cannot be con­strained to be mechanically stable during image acquisition.

Here we demonstrate the application of a spatiotemporal constraint in dynamic CDI, through simulations of imaging an object in motion using realistic experiment parameters relevant to a synchrotron microprobe beamline. The novel aspect of the current work shows that spatiotemporal data redundancy can be used to resolve the twin-image problem. We also explore the relationship between detector frame rate and the observable dynamic behaviour, and how the interplay between signal-to-noise ratio and the amount of spatiotemporal diversity within the system affects the reconstructed image quality. We show that, with sufficient redundancy, it will be possible to observe nanoscale dynamic behaviour at kHz frame rates using existing light sources.

### 1.1. Method

**Figure 1:**
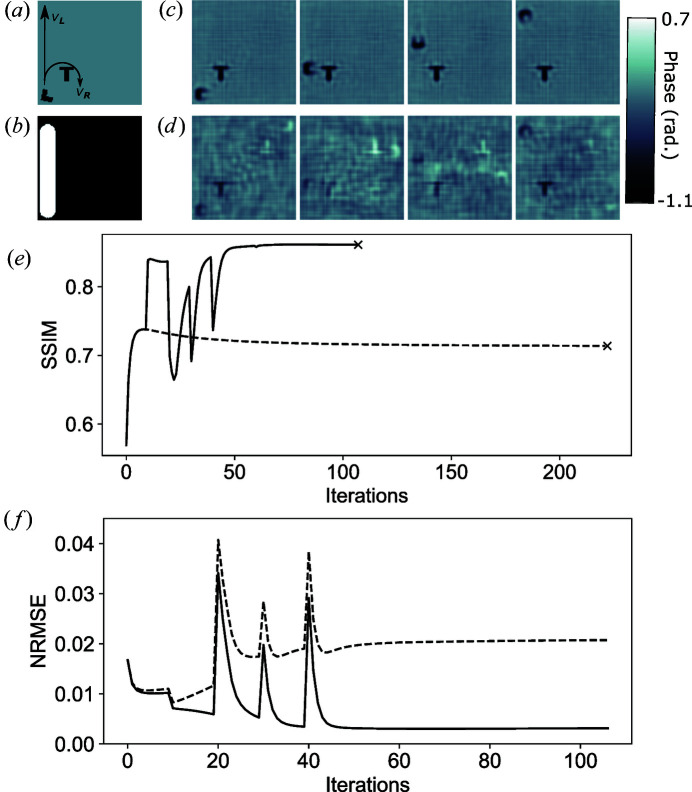
Phase images of a dynamic object: (*a*) indicates the different rotational ( 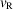 ) and translational ( 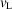 ) behaviour of the L object; (*b*) shows the reconstructed map of dynamic behaviour identifying time-dependent (white) and time-independent (black) areas; phase images for four different time points at a detector frame rate of 0.8 kHz reconstructed (*c*) using a spatiotemporal constraint and (*d*) using the standard CDI algorithm; (*e*) the SSIM compares the reconstruction quality of standard CDI (dashed line) with the spatiotemporal constraint method (solid line), where the point of convergence for both approaches is indicated by a black cross; (*f*) the NRMSE shows the relative error between the reconstructed object (solid) and its twin image (dashed) from the simulated object.

In order to demonstrate this capability, we simulated a synchrotron time-series CDI experiment, with realistic beam intensity and detector parameters. A toy model sample composed of asymmetric moving and stationary objects was chosen, to (*a*) demonstrate the ability of our algorithm to automatically identify dynamic regions of the image throughout time-series CDI; (*b*) highlight the effect of detector frame rate on the observable dynamics and reconstruction quality; and (*c*) make obvious the occurrence of twin images in the reconstructions. A dynamic L-shaped object slides across the field of view with translational speed 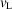 and rotational speed 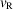 nearby a stationary T-shaped object. The simulated motion of the L is illustrated in Fig. 1 ▸(*a*). The side length of the L and T is *d* = 0.5 µm and the thickness along the beam direction is *t* = 1.0 µm. The photon energy was chosen to be 7.374 keV and the material of the object Fe, for which the complex X-ray refractive index 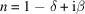 components are 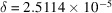 and 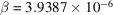 (Henke *et al.*, 1993 ▸). A flat symmetrical probe beam with dimensions 3.5 × 3.5 µm was used to improve the chance of the twin image being reconstructed. For each object frame, the complex exit surface wave ψ was generated under the projection approximation, *i.e.* the projected refractive index distribution represents the amplitude and phase effect of the object on the incident wave. The simulated data are diffraction patterns of the object, obtained by propagating ψ to the detector plane 3.8 m downstream using a far-field (Fraunhofer) propagator and obtaining the intensity with Poisson noise added. Using a detector area of 256 × 256 pixels of size 75 × 75 µm, the diffraction patterns were ‘oversampled’ relative to the smallest speckle size by a factor of approximately 2.4. The pixel size in the reconstructed complex object function was 33 nm. The intensity of the beam was 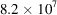 photons s^−1^, which resulted in 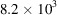 photons per diffraction pattern at a detector frame rate of *f*_0_ = 10 kHz. A time-series data set of *n* = 4800 frames was produced, corresponding to an interval of 0.48 s, during which the L rotates at 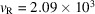 rad s^−1^ and moves across the image field at a speed of 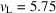 µm s^−1^.

The effect of various frame rates, *f*, on the observable dynamics was investigated by integrating over successive blocks of 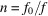 frames, at which point Poisson noise was included. For example, to simulate *f* = 0.4 kHz, the data were integrated in blocks of 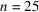 frames, each corresponding to a time interval 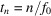 seconds. The signal-to-noise ratio (SNR) within an integrated diffraction frame was then calculated as SNR = 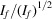 , where 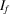 is the mean intensity within an integrated diffraction pattern for a given frame rate. Twelve different frame rates were investigated, from *f* = 0.042 kHz to *f* = 5 kHz.

The 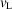 and 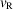 motion of the L can be represented as signals in order to define a critical sampling rate, 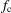 , which is required to resolve the dynamic behaviour according to Nyquist–Shannon theory. To resolve the translational dynamics we must therefore image at a rate 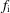 greater than twice the critical frequency, *i.e.* 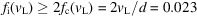 kHz. To re­solve the rotational dynamics, 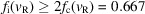 kHz.

The reconstruction of each frame was carried out using the procedure presented by Hinsley *et al.* (2020 ▸), with both spatiotemporally constrained and standard CDI algorithms. The probe profile was assumed to be known, and the number of photons for each *f* was scaled from the photon count within the integrated diffraction patterns. As the ground-truth complex object is known for each frame in the time series, the structural similarity index measure (SSIM) (Wang *et al.*, 2004 ▸), averaged over the time series, was used to track the progress of the reconstructions. Index values between 0 and 1 indicate the similarity of the reconstructed object to the known object, with 1 representing perfect reconstruction. Reconstructions were considered to be converged when the difference between the time-series-averaged SSIM remained below 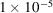 for five consecutive iterations. The normalized root-mean-square error (NRMSE) in real space (Guizar-Sicairos & Fienup, 2012 ▸), averaged over the time series, was used to track the relative weighting between the original ground-truth object and the twin; this measure is invariant to constant and linear phase offsets which can occur within a CDI reconstruction. An NRMSE value of 0 indicates perfect agreement between the image and the ground-truth object. To compare the standard and spatiotemporally constrained CDI reconstructions, the time-series averages of SSIM and NRMSE at convergence were used as error metrics.

## 2. Results and discussion

Fig. 1 ▸ shows reconstructed phase images at four representative time points for *f* = 0.8 kHz. Reconstructions using a spatiotemporal constraint are shown in Fig. 1 ▸(*c*) and using a standard CDI reconstruction approach in Fig. 1 ▸(*d*). Fig. 1 ▸(*e*) indicates that the reconstruction quality is improved when using a spatiotemporal constraint, compared with the standard reconstruction approach. A map of dynamic regions was formed during the reconstruction process by 3D Gaussian smoothing the phase of the current iterate over the spatial and temporal dimensions, shifting the mean phase to be zero, calculating the standard deviation at each position in the object plane over time, and segmenting the result using Otsu’s method (Otsu, 1979 ▸) within the FWHM defined by the probe. This result was then dilated to ensure encapsulation of the entire dynamic region. The map of dynamic regions was updated every ten iterations thereafter using the same process, but the standard deviation through time was segmented using a threshold value of 25% of the maximum value. The threshold value was empirically determined and is able to accurately segment the dynamic behaviour of the object from noise. The map of dynamic regions [Fig. 1 ▸(*b*)] was then used to apply a spatiotemporal constraint within the time-independent region (black), enforcing consistency and using redundant information to improve the image reconstruction across the time series. During the early iterations the map of dynamic regions identified the dynamics of the twin image along with that of the original, but the spatiotemporal constraint caused the twin to be slowly suppressed over subsequent iterations. The final refined map of dynamic regions contains only the dynamic information of the original object. This is also reflected in the NRMSE in Fig. 1 ▸(*f*), which shows the twin and original start with a similar weighting in real space. With application of the spatiotemporal constraint, the NRMSE for the twin increases while the original decreases.

**Figure 2:**
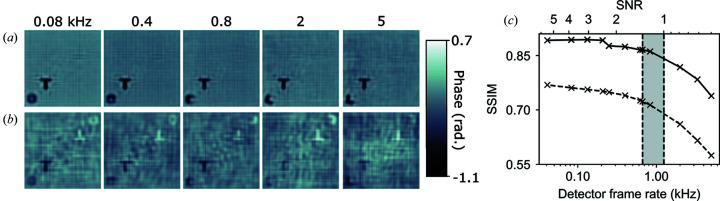
Phase images for detector frame rates from *f* = 0.08 kHz to *f* = 5 kHz: reconstructions (*a*) using a spatiotemporal constraint and (*b*) using the standard CDI algorithm; (*c*) the SSIM compares the reconstruction quality of standard CDI (dashed line) with the spatiotemporal constraint method (solid line), where the shaded region indicates the optimal range of experimental imaging rates 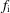 , delimited by twice the rotational critical sampling frequency 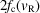 = 0.667 kHz (lower axis intercept) and SNR = 1 (upper axis).

Fig. 2 ▸ shows the effect of detector frame rate, *f*, on the ability to capture dynamics. Phase images for the first time point for different *f* values reconstructed using a spatiotemporal constraint are shown in Fig. 2 ▸(*a*) and using a standard CDI approach in Fig. 2 ▸(*b*). The spatiotemporal constraint refined during the reconstruction process was identical to that shown in Fig. 1 ▸(*b*) for all values of *f*. That is, for each detector frame rate the dynamic behaviour of the original object was correctly identified, and the twin image was suppressed.

At slower detector frame rates 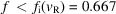 kHz (marked as a vertical dashed line) the rotational dynamics of the L are not temporally resolved and each image in the time series represents the object dynamics averaged over a time interval 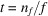 . Consequently, the shape of the L is unrecognizable and it appears as a blurred oval. Where 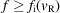 the orientation of the object can be accurately determined for each image in the time series.

Fig. 2 ▸(*c*) shows that the image fidelity is improved for all values of *f* when using a spatiotemporal constraint. The reconstructed image fidelity slowly deteriorates at higher values of *f*. For this simulated object and incident beam intensity the SNR in the diffraction intensity falls below 1 for detector frame rates 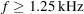 (marked with a dashed line). This coincides with a significant loss of reconstructed image quality and can be taken as an upper limit to 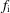 . Taking 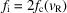 as the lower limit, we can therefore define an optimal experimental range for 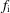 , shown as a shaded region in Fig. 2 ▸(*c*). This rationale can be applied to imaging any dynamic system using an estimate of the critical sampling interval and empirical determination of the value of *f* that corresponds to SNR = 1 in the diffraction intensity.

**Figure 3:**
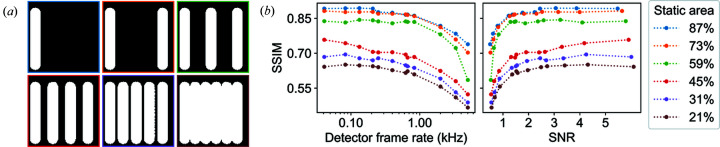
Result of increasing the simulated dynamic behaviour: (*a*) shows simulated spatiotemporal constraint maps illustrating the increase in dynamic behaviour through the addition of independent L objects into the simulation; (*b*) the SSIM compares the reconstruction quality using the spatiotemporal constraint at each level of simulated dynamics, as a function of frame rate and SNR, where the colour of each line corresponds to the borders of the spatiotemporal constraint maps in (*a*).

The effect of the amount of dynamic behaviour on reconstruction quality was investigated by introducing one to five additional dynamic L objects into the simulation. The frame-to-frame behaviour of each L was varied, while the overall dynamic behaviour ( 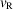 and 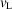 values) across all 4800 frames was kept the same. The simulated map of dynamic behaviour for each test is shown in Fig. 3 ▸(*a*). Reconstructions were carried out using the spatiotemporally constrained approach, with identical parameters in each trial.

The SSIM was used to track the convergence, shown in Fig. 3 ▸(*b*) as a function of *f* and SNR. When the simulated spatiotemporal diversity in the system becomes too high, the time-independent region is reduced to the point where the redundant information is insufficient to resolve the twin-image problem. This can be seen more clearly in the transition between three and four simulated L objects, which corresponds to a change from 59 to 45% static region, respectively. The trend in SSIM as a function of *f* remains identical in each trial, showing that the effectiveness of the incorporation of a spatiotemporal constraint is insensitive to the observed frame-to-frame behaviour and only depends on the overall dynamic behaviour across the time-series data set. The limit on the effectiveness is therefore dependent on the degree of spatiotemporal diversity within the data set and is most effective when there is less than 50% diversity within the image field of view. This is compatible with the characteristics of a wide range of *in situ* studies, including nucleation growth/dissolution of nanostructures (Sun & Wang, 2011 ▸; Kuo *et al.*, 2011 ▸), soft-matter dynamics (Auernhammer *et al.*, 2009 ▸) and real-time monitoring of additive manufacturing (Zhao *et al.*, 2017 ▸).

Applying this method to systems with higher spatiotemporal diversity requires future work: designing an improved approach to segmenting dynamic regions from time-independent regions, or division of the data into subsets that each contain less spatiotemporal diversity. Note that in a realistic experiment the probe profile may not be symmetrical as has been simulated in this work and may break the twin-image ambiguity. In such cases, the ability to image dynamic systems at kHz frame rates is only limited by the SNR within a diffraction pattern and the effectiveness of the approach in mapping the spatiotemporal dynamics.

## 3. Conclusion

We have demonstrated that the spatiotemporally redundant information obtained over a series of measurements of diffraction intensity using fast detectors can be exploited to reliably overcome image reconstruction ambiguities across the time-series image reconstruction. By taking advantage of developments in detector technology which offer high frame rates, and the development of brighter light sources, our approach can be applied to overcome issues of stability and be used for the investigation of dynamic phenomena which would be unobtainable through methods such as ptychography. For systems in which the spatiotemporal diversity is sufficiently low, the application of this constraint could be used to reliably reconstruct CDI data collected at kHz frame rates.
